# Memories people no longer believe in can still affect them in helpful and harmful ways

**DOI:** 10.3758/s13421-022-01328-9

**Published:** 2022-06-14

**Authors:** Ryan Burnell, Robert A. Nash, Sharda Umanath, Maryanne Garry

**Affiliations:** 1 School of Psychology, University of Waikato, Private Bag 3105, Hamilton, 3240 New Zealand; 2 School of Psychology, Aston University, Birmingham, UK; 3 Department of Psychological Science, Claremont McKenna College, Claremont, CA USA

**Keywords:** False memory, Memory functions, Autobiographical memory

## Abstract

People can come to “remember” experiences they never had, and these false memories—much like memories for real experiences—can serve a variety of helpful and harmful functions. Sometimes, though, people realize one of their memories is false, and retract their belief in it. These “retracted memories” continue to have many of the same phenomenological characteristics as their believed memories. But can they also continue to serve functions? Across four experiments, we asked subjects to rate the extent to which their retracted memories serve helpful and harmful functions and compared these functions with those served by “genuine” autobiographical memories. People rated their retracted memories as serving both helpful and harmful functions, much like their genuine memories. In addition, we found only weak relationships between people’s belief in their memories and the extent to which those memories served perceived functions. These results suggest memories can serve functions even in the absence of belief and highlight the potential for false memories to affect people’s thinking and behavior even after people have retracted them.

**Supplementary Information:**

The online version contains supplementary material available at 10.3758/s13421-022-01328-9.

A participant in one of our studies recounted a vivid memory of a distressing accident. During some home renovations, he told us, a piece of metal flew into his eye. The doctors explained that his eyesight would be unaffected, but they could not remove the metal. But when this man visited a doctor a few years later, he learned there was no piece of metal in his eye. He realized his memory was completely false; there had been no accident, no trip to the doctor. These false memories—like real memories—can be compelling and affect people’s thinking and behavior in both helpful and harmful ways (Bernstein et al., 2005; Laney et al., 2008; Pillemer, 1992). For example, our participant’s memory might have led him to be more cautious in his home repairs. But even after this man realized the accident never happened, he still retained a vivid “memory” of the event. Could this memory, once retracted, still affect his behavior?

This question is important to answer because this man’s experience is not uncommon. In fact, approximately 20% of adults can think of at least one “memory” of an event they now realize never happened (Mazzoni et al., 2010). These “memories” have been referred to as “nonbelieved memories,” and are typically defined as vivid memories for events people once believed to be true, but which they later stopped believing. Crucially, people still retain the “memory” even though they no longer believe the events happened (Mazzoni et al., 2010; Scoboria et al., 2015). These memories comprise a class of memories of interest for a variety of theoretical and practical reasons (Otgaar et al., 2015).

But are they best described as “nonbelieved memories?” It is true that, as a package, people are no longer willing to call the memories real. Yet people often report belief in at least some aspects of these memories, which does not square with the term “nonbelieved” (Scoboria, Nash, et al., 2017). Of course, this continued belief is hardly surprising in light of work showing false memories typically feature plausible arrangements of people, places, and objects (Hyman & Kleinkneckt, 1999; Mazzoni et al., 2001). Also, the term “nonbelieved memories” does not convey a crucial feature of these memories—that people once believed in the parts of the memory they eventually realized were not real. To address these concerns, we turned to the false memory literature, in which the term “retracted” has long been used to describe memories people used to believe, but no longer do (Lief & Fetkewicz, 1995; Ost, 2017). “Retracted memories” can accommodate memories in which people hold some belief, but which do not, ultimately, meet the threshold to be endorsed as real. Moreover, the term “retracted” echoes usage in legal settings. For all these reasons, we adopt the term “retracted memories” and suggest this term better captures memories people have come to realize are false.

To understand the potential for retracted memories to affect people’s thinking and behavior, we first need to consider the effects of “genuine” memories. The literature suggests that autobiographical memories serve at least three broad helpful functions (Bluck et al., 2005; Pillemer, 1992). First, they *direct* thinking and behavior—for example, the memory of an academic success might lead people to pursue further studies (Addis et al., 2007; Pillemer, 2003). Second, they help people maintain a coherent sense of *self*—for instance, that same academic success might give people confidence that they are intelligent and capable (Conway, 2005; Ross & Wilson, 2003). Third, people share their memories with others to help forge and maintain vital *social* relationships—for example, people might discuss with their family the stress of waiting for their exam results to elicit empathy (Alea & Bluck, 2003). Recent evidence suggests memories can also serve harmful versions of these functions (Burnell et al., 2020). For example, the memory of an academic failure might direct behavior in harmful ways if it leads people to give up on their career and might serve a harmful self function if it leads people to think they are not intelligent. And if people repeatedly complain about this failure to others, it could damage their relationships with those others—a harmful social function. In fact, there is evidence that memories can serve a mix of these helpful and harmful functions (Burnell et al., 2020). Could retracted memories still serve these functions, helpful and harmful, even after people have retracted them? That is the question we address here.

On the one hand, there are reasons to think retracted memories might not continue to serve functions. From an evolutionary perspective, it would not be particularly adaptive for people to rely on memories they know are wrong (Nairne et al., 2008; Scoboria et al., 2017a). After all, it has been suggested that a key evolutionary advantage of memory is that it enables organisms to remember past experiences and adjust subsequent behavior accordingly (Klein et al., 2002). For example, humans’ ability to remember where to find food and water provides obvious survival advantages (Ginsburg & Jablonka, 2010). But if these memories were wrong, relying on them might not be adaptive—there would be little utility in searching for a water source that never existed. In fact, relying on these memories might be maladaptive, leading to unnecessary expenditure of time and energy. Therefore, when people realize one of their memories is false, we might expect them to stop relying on it. There﻿ is some evidence for this idea: In one study, subjects were led to falsely remember reading a “lure” word from a Deese–Roediger–McDermott list (Deese, 1959; Wang et al., 2017; Roediger & McDermott, 1995). Later, when asked to solve a problem related to the lure word, subjects who believed they saw the lure word tended to be faster at solving the problems than were subjects who never developed this false memory. But more importantly, subjects who initially believed they saw the lure word, but then learned their memory was wrong, did not show this advantage. These findings fit with the idea that when people stop believing in a memory, that memory is less likely to guide their thinking and behavior.

On the other hand, there are reasons to think retracted memories might continue to serve functions. For one thing, when people imagine events that might happen in the future, they clearly do not believe those events have happened. Yet future thoughts have profound effects on thinking and behavior (Daniel et al., 2013; Suddendorf & Corballis, 2007; see also Sanson et al., 2018). Perhaps, then, the vivid phenomenological characteristics of these thoughts help drive them to serve functions. Consistent with this idea, vivid phenomenological characteristics have been linked with the functions of memories. Autobiographical memories often contain valuable perceptual, spatial, and temporal information people can draw on, and which helps cue memories when they are needed (Pillemer, 1992; Schacter & Madore, 2016; Tulving, 1985; Williams, 2007). Therefore, if retracted memories retain these episodic characteristics, they might continue to serve functions. Indeed, when people retract their belief in a memory, many of the characteristics of that “memory”—including the sense of reliving that accompanies it—remain relatively unaffected (Mazzoni et al., 2010; Scoboria et al., 2014). Why? One explanation is that belief is not an inherent or stable property of a memory. Instead, it is an attribution people make in the moment, based on factors such as the phenomenological characteristics of that memory, whether it makes logical sense, and whether it fits with supporting memories (Johnson et al., 1988; Johnson et al., 1993; Sanson et al., 2020; Taylor et al., 2020). Therefore, if belief is simply an attribution, retracting a memory should have minimal impact on the characteristics of that memory. And if these phenomenological characteristics are what drives memories to serve functions, we might expect retracted memories to continue serving functions.

To what extent, then, do retracted memories serve functions? If belief in memories is important for those memories to serve functions, we should expect retracted memories to serve functions less than “believed” memories. But if functions depend at least in part on phenomenological characteristics, retracted memories should continue to serve at least some functions. Across four experiments, we addressed this question by asking people to report the helpful and harmful functions of their retracted memories. We then compared these functions to those served by believed memories.

## Experiment 1

In Experiment 1, we measured the self-reported helpful and harmful functions of people’s retracted memories and compared these functions with those served by memories that people still believe. This experiment was preregistered; the preregistrations, supplemental materials, and data for all experiments reported in this paper are available on the Open Science Framework (https://osf.io/q3sjm/). These experiments were approved by The University of Waikato’s School of Psychology Research Ethics Committee under the delegated authority of the University’s Human Research Ethics Committee.

### Method

#### Subjects

We recruited workers on Amazon’s Mechanical Turk platform (MTurk; https://www.mturk.com/) through TurkPrime (Litman et al., 2017).^1^ Subjects participated in exchange for Amazon credit. To give us at least 80% power to detect medium effects (*d* = 0.4), we sought to collect data from 100 subjects with retracted memories. Expecting 20%–25% of subjects to report having a retracted memory, we aimed to collect data until 400 subjects had completed the survey. Because of the way MTurk interacts with Qualtrics, 414 subjects completed the survey. According to our preregistered criteria, we then excluded 16 subjects who did not provide an autobiographical memory—for example, one subject simply wrote “good.” In addition to these preregistered exclusions, we excluded a further 14 who clearly misunderstood the instruction to provide a retracted memory—for example, several subjects wrote about negative experiences they wish had never happened. Finally, we excluded seven subjects who provided a believed memory outside the requested 4–10 age range,^2^ leaving us with our final sample of 377 subjects (236 women, 138 men, three gender diverse; *M*_age_ = 37.10, *SD*_age_ = 12.34; see the Supplemental Materials for information about level of education).

#### Procedure

First, we provided subjects with a description of a retracted memory, adapted from the literature (Mazzoni et al., 2010):

“Sometimes, people have a memory for an event, but they stop believing the event really happened to them. Nevertheless, their ‘memory’ for the event continues to feel like a real memory.”

Then, we asked subjects whether they have one of these memories. Subjects who said “yes” described that memory. Subjects who said “no” served as our comparison group and described a “believed” memory that occurred between the ages 4 and 10. We used this age range to match the age of the believed memories to the retracted memories, which mostly fell within this range in a landmark study of retracted memories (Mazzoni et al., 2010).

Next, subjects rated their nominated memory on four pairs of items measuring the extent to which a memory is helpful and harmful, such as “This memory guides my thinking and behavior in ways that help me” (Burnell et al., 2020; Rasmussen & Berntsen, 2009). These items appear in Table 1. Then, subjects rated their belief in the memory on a single item and reported how old they were when the event “occurred” (see Table 1). Finally, subjects who described a retracted memory were asked when and why they stopped believing in the memory—because these data are not central to our research question, they can be found on the Open Science Framework.

**Table 1:** Function, belief, and valence items from Experiments 1, 2, and 3

Helpful functions	
This memory guides my thinking and behavior in ways that help me (*1 = Not at all 7 = To a very high degree*)	
This memory tells me about my identity in ways that help me (1 = *Not at all, 7 = To a very high degree*)	
I share this memory with other people in ways that help me (1 = *Not at all, 7 = To a very high degree*)	
This memory gives me a sense of belonging with other people (1 = *Not at all, 7 = To a very high degree*; Experiments 1 & 2 only)	
Harmful functions	
This memory guides my thinking and behavior in ways that hurt me (1 = *Not at all, 7 = To a very high degree*)	
This memory tells me about my identity in ways that hurt me (1 = *Not at all, 7 = To a very high degree*)	
I share this memory with other people in ways that hurt me (1 = *Not at all, 7 = To a very high degree*)	
This memory gives me a sense of disconnection from other people (1 = *Not at all, 7 = To a very high degree*; Experiments 1 & 2 only)	
Belief	
I believe this event really occurred in the way I remember it, and that I have not imagined or fabricated anything that did not occur (1 = *100% imaginary*, 7 = *100% real*)	
Valence (Experiments 2 and 3 only)	
The feelings I experience as I recall the event are positive (1 = *Not at all*, 7 = *Extremely*)	
The feelings I experience as I recall the event are negative (1 = *Not at all*, 7 = *Extremely*)	

### Results

#### Descriptives

We first classified subjects according to whether they reported having a retracted memory. We found that 106 people (28%) reported having a retracted memory, and 271 (72%) reported not having any. Examples of retracted memories include “I remember being chased by geese at my 8th birthday party in a park, but I feel like I may have gotten that memory from a television show” and “I remember really driving my parent’s car when I was a little kid with my younger sister holding the pedals.” In the mean, subjects’ descriptions were 36.19 words long (*SD* = 25.53, *Mdn* = 28, range: 4–127). Examples of believed memories include “When I was 7, a friend accidentally hit me with a metal bat, and I had to go to the hospital to receive stitches” and “I remember my 5th birthday party. We had a piñata, and a ton of people came. I remember it was probably the biggest party I had as a child.” In the mean, subjects’ descriptions were 38.22 words long (*SD* = 24.03, *Mdn* = 34, range: 3–147).

As expected, subjects believed their retracted memories less than the believed memories (see Table 2). This finding, which suggests residual belief in some retracted memories, fits with prior work (Scoboria et al., 2014; Scoboria et al., 2017b). Subjects believed in their retracted memories for a mean of 9.09 years (*SD* = 9.35) before retracting them, which in turn occurred 12.38 years (*SD* = 10.65) prior to the study. The retracted memories also “occurred” at a later age than subjects’ believed memories (*M*_retracted_ = 13.63, *SD* = 10.47, *Mdn* = 10.50, range: 0–59; *M*_believed_ = 6.49, *SD* = 1.76, *Mdn* = 6, range: 4–10).

**Table 2:** Subjects’ belief ratings for each experiment

Experiment	Believed				Retracted				
	*M*	*Mdn*	*SD*	*M*	*Mdn*	*SD*	*M*_diff_	95% CI	*p*
Experiment 1	6.24	7.00	1.24	4.51	5.00	1.94	1.73	[1.33, 2.14]	<.001*
Experiment 2	6.41	7.00	1.07	3.81	4.00	2.06	2.60	[2.17, 3.02]	<.001*
Experiment 3	5.96	7.00	1.42	3.67	4.00	1.99	2.29	[2.02, 2.55]	<.001*
Experiment 4	5.84	6.00	1.16	3.98	4.00	1.50	1.86	[1.67, 2.05]	<.001*

*p < .05

#### Function ratings

**Fig. 1:**
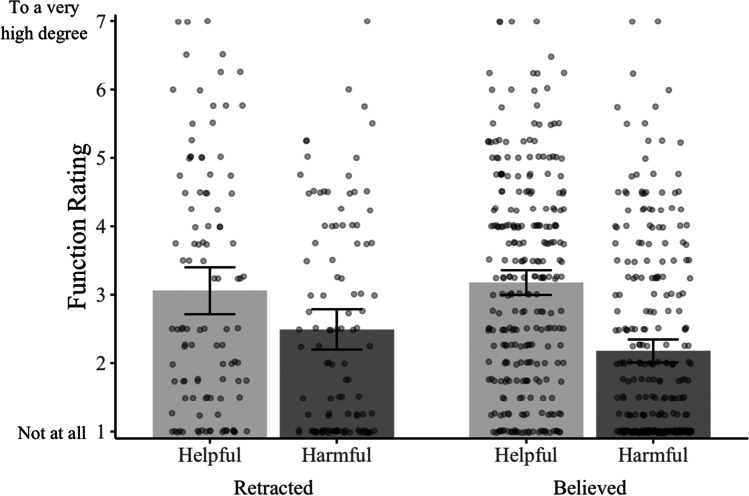
Subjects’ ratings from Experiment 1 of the extent to which their nominated memory serves helpful and harmful functions. Bars represent the mean values; dots (jittered for legibility) represent individual data points. Error bars represent the 95% CIs around the cell means

We now turn to our primary question: To what extent do people think their retracted memories serve helpful and harmful functions? To answer this question, we created a measure of helpful function by taking the mean of the items measuring the helpful directive, self, and social functions for each memory. Likewise, we created a measure of harmful function by taking the mean of the items measuring the harmful directive, self, and social functions. Both measures had good reliability (*α*_helpful_ = 0.85, *α*_harmful_ = 0.88). We display the results of these two measures in Fig. 1—the left side displays subjects’ ratings of their retracted memories, and the right side displays subjects’ ratings of their believed memories. As the distribution on the left side of the figure shows, many subjects rated their retracted memories as at least moderately helpful and harmful. Furthermore, if we compare these ratings to subjects’ ratings of their believed memories, we see the two types of memories were rated as similarly helpful, *M*_retracted_ = 3.06; *M*_believed_ = 3.18; *M*_diff_ = 0.12, 95% CI [−0.27, 0.51], *p* = .547, *d* = 0.07. Likewise, subjects’ believed and retracted memories were rated as similarly harmful, *M*_retracted_ = 2.49; *M*_believed_ = 2.18; *M*_diff_ = 0.32, 95% CI [−0.02, 0.66], *p* = .066, *d* = 0.22. These results suggest many people think their retracted memories continue to serve helpful and harmful functions.

One interpretation of these findings is that people do not need to believe a memory is “real” for it to serve functions. But there is another explanation. As we noted earlier, subjects reported some residual belief in their retracted memories. Perhaps, then, the retracted memories served functions only because subjects still held some belief in them. If so, we would expect the memories subjects believed most to also be the most helpful or harmful. Exploratory analyses provided some evidence for this account. For instance, the more people reported believing in a memory, the more helpful they rated that memory, *r*(104) = .34, 95% CI [0.16, 0.50], *p* < .001. Likewise, the more people reported believing in a memory, the more harmful they rated it, although this relationship was plausibly no different from zero, *r*(104) = 0.17, 95% CI [−0.02, 0.35], *p* = .073. But given our relatively small sample of retracted memories and the wide confidence intervals around these correlations, it is difficult to draw conclusions from them (Schönbrodt & Perugini, 2013).

One limitation of these data is that subjects’ retracted memories tended to be for events that “occurred” at a later age than the believed events. To the extent that recent events might be more vivid and more relevant to people’s current situation, these differences in how long ago the believed and retracted events occurred might be masking differences between believed and retracted memories that would otherwise be apparent. There were two main reasons that subjects’ retracted and believed memories were not well-matched on age. First, subjects tended to provide a believed memory from the lower end of the 4–10 age range—perhaps because our instructions encouraged a search starting at age 4. Second, there was a wider spread in the age distribution of retracted memories than we anticipated based on prior research (Mazzoni et al., 2010; see the Supplemental Materials for a full breakdown). Therefore, we sought to replicate Experiment 1 using believed memories that more closely matched the retracted memories. Experiment 2 was not preregistered but followed the same analytic approach as Experiment 1.

## Experiment 2

### Method

#### Subjects

We recruited workers on MTurk through TurkPrime. Subjects participated in exchange for Amazon credit. We again aimed to collect data until 400 subjects had completed the survey. In total, 414 subjects completed the survey. As in Experiment 1, we excluded 17 subjects who did not provide an autobiographical memory and a further 14 who misunderstood the instruction to provide a retracted memory, leaving us with 383 subjects (235 women, 147 men, one gender diverse; *M*_age_ = 37.08, *SD*_age_ = 12.20).

#### Procedure

The method for Experiment 2 was the same as Experiment 1, with three exceptions. First, given the wide range of belief ratings in Experiment 1, we clarified the definition of retracted memories we provided to subjects:

Sometimes, people have a memory for an event, but they come to realize the event never happened at all—their “memory” was completely false. Nevertheless, their “memory” for the event continues to feel like a real memory, even though they know the event didn’t really happen.

Second, we sought to elicit an age distribution of believed memories that better matched the retracted memories. We asked subjects without any retracted memories to describe a “believed” memory that occurred before the age of 15. Third, recent work suggests negative memories, in particular, are often harmful (Burnell et al., 2020). Therefore, we asked subjects to rate the valence of their memory on two items: one measuring the extent to which the memory elicits positive feelings, and one measuring the extent to which the memory elicits negative feelings (see Table 1).

### Results

#### Descriptives

As in Experiment 1, we classified subjects according to whether they have a retracted memory. In this experiment, 101 people (26%) reported having one, and 282 people (74%) reported not having any. An example of a retracted memory was: “I remember going to a theme park with my family when I was younger and I remember stuff we did but they tell me that we never went anywhere.” Subjects’ descriptions of their retracted memories were 39.55 words long in the mean (*SD* = 22.12, *Mdn* = 34, range: 5–109). An example of a believed memory was: “I remember seeing my grandma the last time before she died hoping it would not be the last.” Subjects’ descriptions of their believed memories were 41.27 words long in the mean (*SD* = 24.73, *Mdn* = 36.50, range: 4–174).

Once again, subjects’ retracted memories were believed less than subjects’ believed memories (see Table 2). Our revised instructions elicited memories that were believed less than in Experiment 1. In the mean, the retracted memories were believed for 7.04 years (*SD* = 7.72) and were retracted 12.67 years ago (*SD* = 11.80). The age distributions were better matched than in Experiment 1, but the retracted memories still occurred later, *M*_retracted_ = 14.99, *SD* = 12.60, *Mdn* = 8, range: 1–55; *M*_believed_ = 10.08, *SD* = 3.36, *Mdn* = 11, range: 1–15.

#### Function ratings

**Fig. 2:**
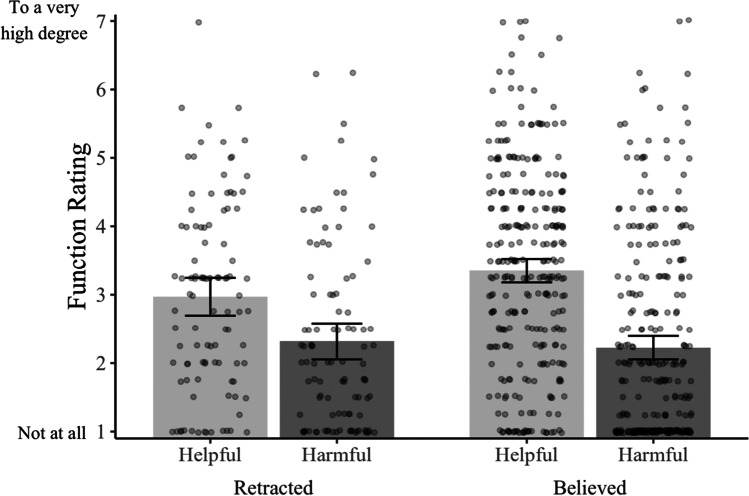
Subjects’ ratings from Experiment 2 of the extent to which their nominated memory serves helpful and harmful functions. Bars represent the mean values; dots (jittered for legibility) represent individual data points, and error bars represent the 95% CIs around the cell means

Next, we returned to our primary research question: to what extent do people think their retracted memories serve functions in helpful and harmful ways? As Fig. 2 shows, we replicated the findings of Experiment 1—many subjects reported their retracted memories serve helpful and harmful functions to at least a moderate degree. We again found no evidence that people think their retracted memories are less harmful than believed memories, *M*_retracted_ = 2.32; *M*_believed_ = 2.23 *M*_diff_ = 0.09, 95% CI [−0.22, 0.40], *p* = .564, *d* = 0.06. We did, however, find that retracted memories were rated as slightly less helpful than believed memories, *M*_retracted_ = 2.97; *M*_believed_ = 3.35; *M*_diff_ = 0.38, 95% CI [0.06, 0.71], *p* = .021, *d* = 0.26. Together, these results provide further evidence that people think their retracted memories continue to serve helpful and harmful functions.

As in Experiment 1, we next examined the relationship between subjects’ belief in their retracted memories and the degree to which those memories serve functions. In this experiment, we found that the relationship between belief and helpful functions was plausibly no different from zero, *r*(99) = 0.16, 95% CI [−0.03, 0.35], *p* = .102. Likewise, the relationship between belief and harmful function was plausibly zero, *r*(99) = 0.07, 95% CI [−0.13, 0.26], *p* = .491. Taken together, we found no compelling evidence to suggest that belief is important for memories to serve functions.

However, subjects’ retracted and believed memories were still not well matched for how long ago the events “occurred.” Therefore, in Experiment 3, we yoked subjects’ believed memories to their retracted memories to ensure the two types of memories were matched on age.

## Experiment 3

So that we could easily yoke subjects’ believed memories to their retracted memories, we switched to a within-subjects design—when subjects reported having a retracted memory, we asked them to describe both that memory and a believed memory that occurred around the same time. In addition, to examine the relationships between the phenomenological characteristics of people’s retracted memories and the functions those memories serve, we added a series of items measuring phenomenological characteristics from the Autobiographical Memory Questionnaire (Rubin et al., 2003, 2004). Finally, we markedly increased our sample size so we could draw stronger conclusions from the correlations between belief and perceived functions. This experiment was preregistered.

### Method

#### Subjects

We recruited workers on MTurk through TurkPrime. Subjects participated in exchange for Amazon credit. We aimed to collect data until 800 subjects had completed the survey to ensure we had a big enough sample of retracted memories to establish stable correlations between belief and functions (Schönbrodt & Perugini, 2013). In total, 827 subjects completed the survey. According to our preregistered criteria, we excluded 40 subjects who failed attention checks, 13 who did not provide an autobiographical memory, and 67 who misunderstood the instruction to provide a retracted memory, leaving us with our final sample of 707 subjects.

#### Procedure

First, we asked subjects if they have any retracted memories, using the same instructions as in Experiment 2. Subjects who said “yes” described that memory and reported how old they were at the time the “event” happened. Those same subjects then described a memory they still believe that happened at the same age. If they could not think of one, we asked them to choose an event that occurred as close to that age as possible. Subjects who did not have any retracted memories rated two believed memories, but because the data from these subjects is not central to our research question, those data can be found on the Open Science Framework.

Next, subjects rated each memory, in counterbalanced order, on a series of scales—completing the full set of scales for one memory before rating the other. First, subjects rated the functions of the memory and their belief in it using the items from Experiments 1 and 2. We removed the function items asking about belonging/disconnection from others because the social belonging function is less agreed upon in the literature (Rasmussen & Berntsen, 2009). Then, subjects completed 17 items measuring the phenomenological characteristics of the memory, such how vivid the memory is, and the extent to which it is accompanied by a sense of reliving (see the Supplemental Materials for the full list of items and the results for each item; Rubin et al. 2003). Finally, subjects reported when and why they retracted the memory.

### Results

#### Descriptives

Once again, we classified subjects according to whether they reported having a retracted memory. A larger percentage of subjects reported having a retracted memory than in the first two experiments: 321 people (45%) reported they have one, and 386 people (55%) reported they do not have any. Because our key comparison was within subjects, we report here only the data from the 321 subjects who reported having a retracted memory (201 women, 115 men, five gender diverse; *M*_age_ = 36.49, *SD*_age_ = 11.40).

In the mean, subjects’ descriptions of their retracted memories were 33.30 words long (*SD* = 21.51, *Mdn* = 29, range: 4–130). Subjects’ descriptions of these believed memories were 25.18 words long in the mean (*SD* = 17.54, *Mdn* = 21, range: 2–109).

As in Experiments 1 and 2, subjects believed their retracted memories less than their believed memories (see Table 2). In the mean, the retracted memories were believed for 5.94 years (*SD* = 7.31) and were retracted 15.88 years ago (*SD* = 12.27). Unlike Experiments 1 and 2, subjects’ believed memories and retracted memories “occurred” at a similar age (*M*_retracted_ = 14.67, *SD* = 12.75, *Mdn* = 10, range: 0–68; *M*_believed_ = 14.61, *SD* = 12.32, *Mdn* = 10, range: 1–68).

#### Function ratings

**Fig. 3:**
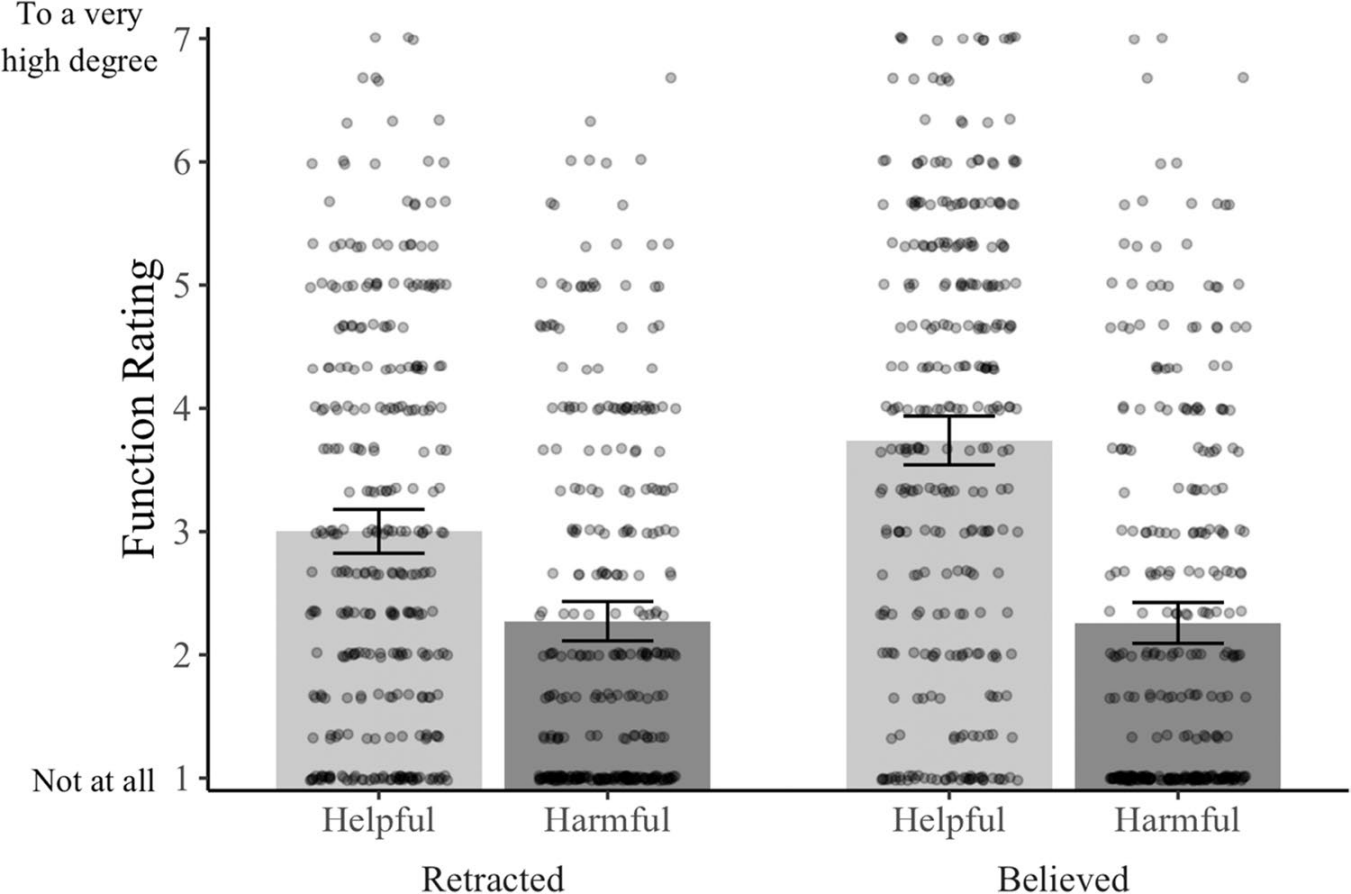
Subjects’ ratings from Experiment 3 of the extent to which their believed and retracted memories serve helpful and harmful functions. Bars represent the mean values; dots (jittered for legibility) represent individual data points, and error bars represent the 95% CIs around the cell means

Now, we return to our primary question. As Fig. 3 shows, we found converging evidence for the idea that many retracted memories serve helpful and harmful functions. In contrast to Experiment 1, but consistent with Experiment 2, subjects rated their retracted memories as less helpful than their believed memories, *M*_retracted_ = 3.00; *M*_believed_ = 3.74; *M*_diff_ = 0.74, 95% CI [0.55, 0.93], *p* < .001, *d* = 0.44. Subjects’ believed and retracted memories were rated as similarly harmful, *M*_retracted_ = 2.27; *M*_believed_ = 2.26; *M*_diff_ = 0.01, 95% CI [−0.13, 0.16], *p* = .848, *d* = 0.01.

These differences in helpful functions once again raise the possibility that belief in a memory is important for that memory to serve helpful functions. Consistent with this idea, we found that the more people believed in a retracted memory, the more helpful it tended to be, *r*(319) = 0.37, 95% CI [0.27, 0.46], *p* < .001. We also found that that the more people believed in a retracted memory, the more harmful it tended to be, although this relationship was weaker, *r*(319) = 0.14, 95% CI [0.03, 0.25], *p* = .011. These results suggest belief might be related to the functions memories serve.

#### The contribution of phenomenology

There is an important counter-explanation, though. Because of our design, we could not manipulate belief independently of other characteristics. We found that subjects’ retracted memories were also different from believed memories in phenomenology—consistent with previous work (Mazzoni et al., 2010). For example, retracted memories were less vivid than believed memories, *M*_retracted_ = 4.92; *M*_believed_ = 5.46; *M*_diff_ = 0.54, 95% CI [0.36, 0.72], *p* < .001, *d* = 0.36, and were accompanied by a less intense sense of reliving, *M*_retracted_ = 4.47; *M*_believed_ = 5.04; *M*_diff_ = 0.56, 95% CI [0.38, 0.75], *p* < .001, *d* = 0.35. To fully understand the relationship between belief and functions, it is important to also consider the contribution of phenomenology.

Therefore, we conducted exploratory regressions to investigate how the functions of retracted memories are related to belief and two key components of phenomenology: reliving and vividness (Rubin et al., 2019). We conducted two linear regressions using the data from subjects’ retracted memories: one with belief, vividness, and reliving predicting helpful function, and one with belief, vividness, and reliving predicting harmful function. As Table 3 shows, belief and reliving were independently related to the helpful functions served by subjects’ retracted memories, such that the more people believed in a memory, and the more it was accompanied by a sense of reliving, the more helpful people rated it. In addition, belief was related to harmful functions such that the more people believed in a memory, the more harmful they rated it—although the size of this effect is plausibly very small. These results are consistent with the hypothesis that belief is important for memories to serve functions and suggest that a sense of reliving might also be important—at least for helpful functions.

**Table 3:** Standardized Beta estimates from the regressions from Experiment 3 predicting helpful and harmful functions among subjects’ retracted memories

	Reliving		Vividness		Belief	
Dependent Measure	*β* [95% CI]	*p*	*β* [95% CI]	*p*	*β* [95% CI]	*p*
Helpful Function	0.21 [0.09, 0.32]	.001*	0.09 [−0.04, 0.21]	.162	0.27 [0.19, 0.36]	<.001*
Harmful Function	0.03 [−0.08, 0.15]	.635	0.04 [−0.09, 0.16]	.589	0.12 [0.03, 0.20]	.045

*p < .05

Given we found no evidence that phenomenology is related to harmful functions, what other factors might drive these functions? One recent study found evidence of harmful functions only in negative memories, a finding that might suggest the valence of a memory is important (Burnell et al., 2020). Because a fair number of the retracted memories in this study were negative—30.53% were rated above the midpoint on the item assessing negative feelings—we conducted exploratory correlations between negative feelings and harmful functions. We found that the more a memory elicited negative feelings, the more harmful it was rated, *r*(319) = 0.42, 95% CI [0.33, 0.51], *p* < .001. Furthermore, adding negative feelings to the regression model predicting harmful functions showed that this relationship held even after accounting for belief, reliving, and vividness (p < .001; see the Supplemental Materials for the regression table). By contrast, the relationship between negative feelings and helpful functions was trivial, *r*(319) = 0.04, 95% CI [−0.07, 0.15], *p* = .448. These findings suggest that characteristics specific to negative memories, such as the emotions those memories elicit or the kinds of events that these memories depict might be important for memories to serve harmful functions.

Taken together, these three experiments provide some evidence that belief is related to the helpful functions a memory serves—or, at least, to the functions people think the memory serves. But this possibility merits further investigation, for several reasons. First, the regression analyses we conducted in this experiment were exploratory. Second, we have so far used only single-item measures of belief, reliving, and vividness that might not capture all aspects of those constructs. Third, we have so far treated helpful functions as one unitary construct. But it is possible the relationships between belief and functions might be different for different functions. For example, people might not draw on retracted memories when making decisions but might still talk about those memories with others to forge relationships—after all, people often embellish or lie about their experiences when talking to others (Marsh & Tversky, 2004).

Therefore, in Experiment 4, we conducted a more robust, preregistered investigation of the relationships between belief, reliving, and helpful functions. To ensure we gathered memories with a wide enough range of belief to see meaningful relationships, we broadened our memory prompt instructions—rather than asking people for a memory they have completely retracted, we asked them for the memory about which they have the most doubts.^3^

## Experiment 4

Experiment 4 departed from the previous experiments in several ways. First, rather than asking for retracted memories, we asked people for memories they doubt. Second, we employed multi-item measures of belief, reliving, and vividness adapted from the Autobiographical Memory Questionnaire (see Rubin et al., 2019). Third, to better enable us to separately examine directive, self, and social functions, we used a different measure of functions: the Thinking About Life Events (TALE) questionnaire, which includes five items measuring each of the directive, self, and social functions (Bluck & Alea, 2011). This experiment was preregistered.

### Method

#### Subjects

We recruited workers on MTurk through TurkPrime. Because precision around the effect sizes from Experiment 3 was good (see Cumming, 2014), we aimed for a similar sample size in this Experiment. We anticipated most subjects to be able to think of a memory they doubt, so we aimed to collect data until 400 subjects had completed the survey. In total, 413 subjects completed the survey. According to our preregistered criteria, we excluded 13 subjects who failed both attention checks, 90 subjects who failed to provide two autobiographical memories, and 14 subjects who reported they could not think of a memory they doubt, leaving us with our final sample of 298 subjects.

#### Procedure

First, we provided subjects with a description of a doubted memory: “Sometimes people have doubts about particular memories of their past experiences—that is, they doubt whether the events they remember really happened at all.”

Then, we asked subjects to describe the memory they have the most doubts about. As a comparison, we asked subjects to describe a believed memory that happened to them during their early childhood.^4^

Next, subjects rated the functions, belief, and phenomenology of each memory. Subjects made these three sets of ratings in counterbalanced order and completed all ratings for one memory before rating the other.

##### Functions

Subjects rated the functions of their memory on the TALE (Bluck & Alea, 2011). We adapted this questionnaire to ask about a specific memory (see the Supplemental Materials). In this scale, subjects complete 15 items that ask about how often they think or talk about the memory for a series of reasons, on a scale from 1 (*almost never*) to 5 (*very frequently*). Five of the items ask about reasons that map on to the directive function, five about reasons that map onto the self function, and five about reasons that map onto the social function. Note that this scale measures only helpful functions—we did not include a measure of harmful functions because in Experiment 3 belief was only a weak predictor of harmful functions and we found no evidence that vividness or reliving predicts harmful functions.

#### Belief

Subjects rated their belief in the memory on the three items from Rubin et al. (2019). The first of these items was the belief item from Experiments 1–3. The second was “My memory of the event is an accurate reflection of the event as a neutral observer would report it and is not distorted by my beliefs, motives, and expectations” (1 = *100% distorted*, 7 = *100% accurate*). The third was “Would you be confident enough in your memory of the event to testify in a court of law?” (1 = *Not at all*, 7 = *As much as any memory*).

#### Phenomenology

Subjects rated the sense of reliving produced by the memory using three items from Rubin et al. (2019), all rated from 1 (*not at all*) to 7 (*as if it were happening now*): “While remembering, it is as if I am living the occurrence again.”; “While remembering, it is as if I am mentally traveling back to the time and place of the occurrence”; and “While remembering, it is as if I am experiencing the same feelings, emotions, and/or atmosphere again.” Subjects rated the vividness of the memory using two items, also from Rubin et al., on a scale from 1 (*not at all*) to 7 (*as vivid as if it were happening now*): “While remembering, I can see everything in my mind.” and “While remembering, the actions, objects, and/or people that are involved in the memory are as clear now as they were when the event occurred.”

### Results

#### Descriptives

We first checked that subjects’ doubted memories were rated lower on belief than their “believed” memories from a similar time. They were (see Table 2). Examples of subjects’ doubted memories include “I have a memory about my learning to ride a bike. I remember my dad teaching me to ride the bike when I was 5 years old, but my aunt said that my dad was out of town and she taught me. My aunt has been known to stir up trouble so I am not really sure if I can believe her. My dad said that he taught me” and “I have doubts about my memory of my grandmother’s death.”

#### Function ratings

**Fig. 4:**
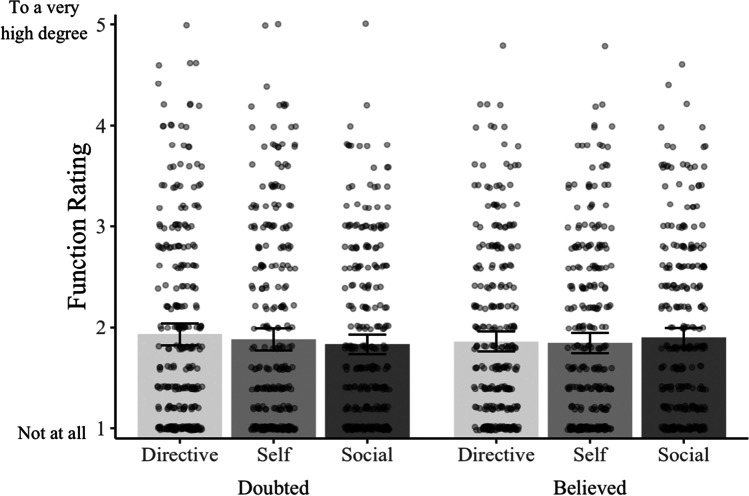
Subjects’ ratings from Experiment 4 of the extent to which their believed and doubted memories serve helpful and harmful functions. Bars represent the mean values; dots represent individual data points, and error bars represent the 95% CIs around the cell means

Before we addressed our primary aim, we next examined subjects’ ratings of the functions served by their doubted memories. Recall that in this experiment, we used a function scale that measures only the degree to which a memory is helpful. We created a sum variable for each of the directive, self, and social functions by taking the mean of the five items from the TALE measuring that function. We display these function sum variables for the doubted and believed memories in Fig. 4. As the figure shows, subjects rated the functions of the two types of memory as remarkably similar—we found no difference between doubted and believed memories in terms of directive functions, *M*_doubted_ = 1.93, *M*_believed_ = 1.86, *M*_diff_ = 0.07, 95% CI [−0.03, 0.17], *p* = .156, *d* = 0.08; self functions, *M*_doubted_ = 1.88, *M*_believed_ = 1.84, *M*_diff_ = 0.04, 95% CI [−0.07, 0.14], *p* = .478, *d* = 0.04; or social functions, *M*_doubted_ = 1.83, *M*_believed_ = 1.90, *M*_diff_ = 0.07, 95% CI [−0.03, 0.17], *p* = .169, *d* = 0.08. These results replicate the previous experiments, and further support the idea that memories can serve functions even when people doubt their veracity.

#### Predicting functions from belief, reliving, and vividness

Next, we addressed our primary aim: to investigate the relationships among belief, recollection, and self-reported helpful functions. To do so, we created a sum variable for belief by calculating the mean of the three items measuring belief, a sum variable for reliving by calculating the mean of the three items measuring reliving, and a sum variable for vividness by calculating the mean of the two items measuring vividness. Then, for each type of memory (doubted and believed), we conducted three preregistered linear regressions—one for each function—with belief, vividness, and reliving as predictors.

We first consider the results from the regressions conducted on subjects’ ratings of their doubted memories, displayed in the top half of Table 4. As the table shows, reliving predicted the directive and self functions of subjects’ doubted memories, such that the more a memory was accompanied by a sense of reliving, the more it tended to serve those two functions. There was again no evidence that vividness was related to the functions of these doubted memories. These findings are consistent with the pattern we found in Experiment 3. But in contrast to Experiment 3, we found no evidence that belief predicted the functions of doubted memories.

**Table 4:** Standardized Beta estimates for the regressions from Experiment 4

Dependent Measure	Reliving		Vividness		Belief	
	*β* [95% CI]	*p*	*β* [95% CI]	*p*	*β* [95% CI]	*p*
Doubted Memories						
Directive function	0.14 [0.02, 0.25]	.018*	−0.03 [−0.14, 0.07]	.550	0.09 [−0.03, 0.20]	.138
Self function	0.13 [0.02, 0.25]	.026*	−0.02 [−0.13, 0.08]	.650	0.05 [−0.07, 0.16]	.441
Social function	0.02 [−0.08, 0.12]	.669	0.05 [−0.04, 0.14]	.284	−0.01 [−0.11, 0.10]	.910
Believed Memories						
Directive function	0.14 [0.04, 0.25]	.007*	0.07 [−0.04, 0.17]	.231	−0.18 [−0.31, −0.05]	.007*
Self function	0.21 [0.11, 0.31]	<.001*	0.02 [−0.08, 0.13]	.665	−0.11 [−0.24, 0.02]	.084
Social function	0.05 [−0.06, 0.15]	.372	0.09 [−0.02, 0.20]	.120	−0.14 [−0.27, −0.01]	.041*

*p < .05

We next consider the regressions conducted on subjects’ ratings of their believed memories, displayed in the bottom half of Table 4. Here, too, we found that reliving predicted the directive and self functions, such that the more a believed memory was accompanied by a sense of reliving, the more it tended to serve those functions. We also found that belief predicted the directive and social functions of subjects’ believed memories, such that the more subjects believed in a memory, the less it tended to serve these functions. This pattern of results is the opposite of what we would expect if belief in a memory is important for that memory to serve functions—and provides some evidence against this hypothesis.

Taken together, we did not find evidence to suggest that belief is important for memories to serve functions. Instead, the most consistent finding from these regression analyses was that the more a memory was accompanied by a sense of reliving, the more people rated it as serving directive and self functions.

## Meta analyses

Across the four experiments, our findings about the relationships between belief and functions were inconsistent. For example, in Experiment 1 we found a moderate positive correlation between belief and helpful function, but in Experiment 2 that relationship was smaller and plausibly zero. Therefore, to obtain more precise estimates of the strength of these relationships, we followed recommendations from Cumming (2014) and conducted two exploratory (not preregistered) mini meta-analyses (see also Goh et al., 2016).

**Fig. 5:**
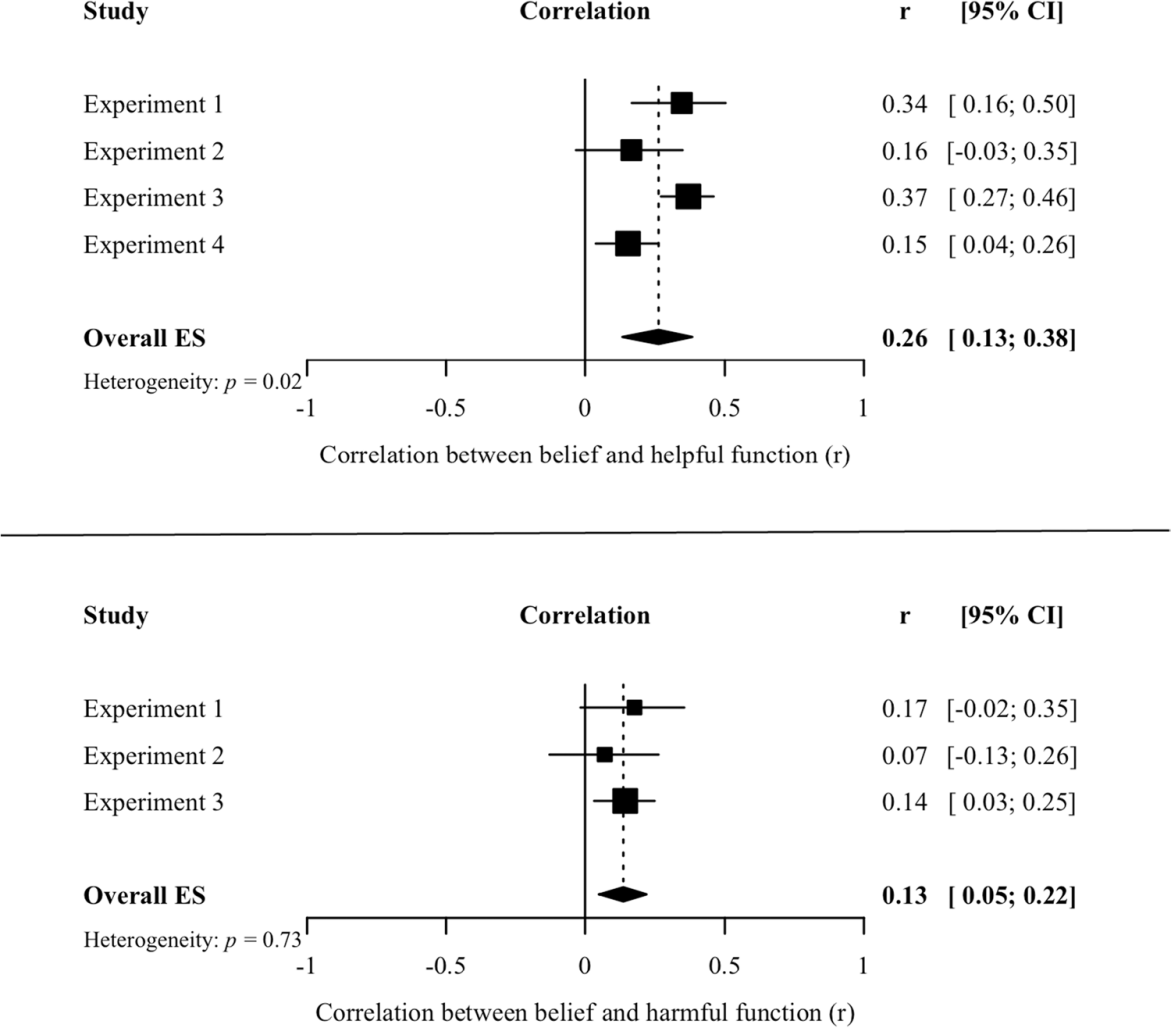
Forest plots of the relationships between belief and functions. The top panel displays the forest plot of the correlations between belief and helpful functions. The bottom panel displays the forest plot of the correlations between belief and harmful functions. Squares represent the correlations for each experiment, diamonds represent the overall effect size estimate from the meta-analyses

First, we conducted a meta-analysis of the relationships between belief and perceived helpful functions across the four experiments. As the top panel of Fig. 5 shows, this analysis suggested there was a small, positive correlation between belief and helpful functions. Next, we conducted a meta-analysis of the relationships between belief and harmful functions across the first three experiments (we did not measure harmful functions in Experiment 4). As the bottom panel of Fig. 5 shows, we also found a small positive relationship between belief and harmful functions. Given that the four experiments differed in some aspects of their design and there was some heterogeneity in the results, the overall effect size estimates should be considered with some caution. But across the board, the results suggest belief is, at best, weakly related to the functions people think their memories serve.

## General discussion

Across four experiments, we investigated the extent to which retracted memories serve functions. We consistently found that people think their retracted memories serve helpful and harmful functions and found only weak relationships between people’s belief in a memory and the reported functions of the memory. We also found in both Experiments 3 and 4 that the more a memory was accompanied by a sense of reliving, the more helpful it tended to be.

Taken together, our findings do not support the hypothesis that belief is important for memories to serve functions. If belief were important, the memories people have retracted should serve functions less than the memories they still believe. But we instead found that people think their retracted memories continue to serve functions to a similar degree as believed memories. In addition, mini meta-analyses showed that belief was, at best, only weakly related to helpful and harmful functions. Moreover, in Experiment 4, these relationships did not hold after controlling for vividness and sense of reliving. It seems likely that a range of factors are important for memories to serve functions (see, e.g., Lind et al., 2019). But considered together, our data fit with the idea that believing a memory is “real” is not a prerequisite for that memory to affect people’s thinking and behaviour.

Our findings do, however, support the hypothesis that the phenomenology of memory plays a role in at least some memory functions—we consistently found relationships between a sense of reliving and ratings of helpful self and directive functions, even after controlling for subjects’ belief in the memory. These findings are consistent with other literatures showing that both thoughts about the future and counterfactuals about the past can evoke emotions and influence behavior, even though people do not believe those events have happened (Daniel et al., 2013; Rasmussen & Berntsen, 2013; Roese, 1994). Given that autobiographical memories tend to be accompanied by a greater sense of reliving than other kinds of memories (e.g., vicarious memories), this finding could partly explain why people place high importance on their own experiences (Pillemer et al., 2015). These findings also extend work investigating the relationships between memory characteristics (Rubin et al., 2019). But it remains unclear why reliving was not related to the helpful social function given that emotions and perceptual details play an important role in communication (Pillemer, 1992; Rimé et al., 1992). Moreover, we found no evidence that memory phenomenology plays a role in harmful functions—neither reliving nor vividness predicted ratings of harmful functions in Experiment 3. Yet in more extreme negative memories, we might see a different pattern—after all, reliving traumatic memories in vivid detail is a hallmark symptom of maladaptive responses such as posttraumatic stress disorder (American Psychiatric Association, 2013). Regardless, our results raise the possibility that helpful and harmful functions are driven by different factors. If so, one interpretation of this pattern is that helpful and harmful functions are not entirely parallel and might be better thought of as distinct consequences of memory—albeit with conceptual similarities.

It is reasonable to wonder, though, how well self-reported functions correspond to the “true” functions served by people’s memories. For example, did subjects understand the broad function items from the first three experiments, such as the one measuring the extent to which a memory “guides my thinking and behavior in ways that help me”? The data suggest they did—the findings from the experiments using these broad items are consistent with those of fourth experiment, which measured functions using more concrete, granular items (e.g., “I think or talk about this memory when I want to try to learn from my past mistakes”). A related issue is that rating the functions of a memory is a complex metacognitive task. Although this approach is commonly used in the literature, it requires subjects to bring to mind occasions during which they had previously thought about the memory and then to evaluate how the memory affected their thinking and behavior on those occasions (Bluck & Alea, 2011). Because of the complexity and retrospective nature of this task, a better approach might be to experimentally implant false memories and then measure the effects of those memories on specific behaviors after the participants learn the memories are false. For instance, in one study, subjects were led to believe they loved asparagus the first time they tried it (Laney et al., 2008). This false memory influenced subjects’ behavior, leading them to rate asparagus as more appealing than subjects without the false memory. Researchers could use this paradigm to measure the extent to which these effects continue after subjects learn their asparagus memory is false. Such an experiment would provide a stronger test of how important it is for people to believe a memory for that memory to serve functions.

These experiments investigated how the characteristics of a memory are related to the functions served by that memory. But it is also important to investigate how functions are related to the characteristics of the person recalling the memory. For example, some people place more importance on their memories than others, and people’s emotion regulation strategies during recall vary markedly (see Rubin et al., 2011). More importantly, these tendencies are related to how people respond following traumatic events. Therefore, we might expect to see individual differences in the extent to which memories are helpful or harmful—that is, two people with similar memories might be affected by those memories in different ways (Bonanno, 2004).

Of course, these experiments have limitations. First, people’s retracted and believed memories differed not only in belief, but in phenomenology—for example, believed memories were accompanied by a stronger sense of reliving. We attempted to address this limitation by controlling for reliving and vividness in our regression models, but the differences in phenomenology between retracted and believed memories made it difficult to isolate the effects of belief. Second, we asked people only about the functions their memories currently serve. It is therefore impossible to know with any degree of precision how, if at all, people’s memories changed when they were retracted. These memories might continue to serve the same functions as they did before the retraction. But it is also possible that some functions have reduced, while new functions arose—for example, people might have stopped relying on the memory itself when making decisions but might now be less trusting of their memories in general.

Our findings also resonate with the idea that memories do not need to be faithful representations of personal experiences in order be useful (Johnson & Sherman, 1990). Indeed, people sometimes draw on both memories of fictional stories and “vicarious memories” of other people’s experiences when making decisions (Bandura, 2006; Pillemer et al., 2015; Yang, 2018). And even among people’s own autobiographical memories, the objective truth of what happened is not always important. When people share their memories with others, they often tweak, embellish, or lie about their experiences to engage or impress those listening (Marsh & Tversky, 2004; McCann & Higgins, 1992). Furthermore, the way people remember and interpret past events changes over time depending on their circumstances and goals (Conway, 2005; Johnson & Sherman, 1990). Some memory distortions might even be adaptive. For example, when considering memories of successes and failures from a similar time, people tend to judge the failures as further in the past—a tendency that might help people maintain a positive view of their current self (Wilson & Ross, 2003). The data reported here bolster these ideas by providing evidence that memories might serve functions even after being retracted.

To the best of our knowledge, this study is the first to investigate the effects of real-world retracted memories on people’s thinking and behavior. We have long known that false memories can have a range of helpful and harmful effects, but our findings suggest these effects might continue even after the memories are identified as false (Bernstein et al., 2005; Laney et al., 2008). In doing so, our results highlight the potential for false memories to do both lasting good and lasting harm. For this reason, one could interpret our findings as evidence that studies that implant false memories have the potential to produce memories that are harmful to subjects even after debriefing. But there are a number of reasons to think such a scenario is unlikely. First, false memory studies do not implant the extremely negative memories that our data suggest are most likely to be harmful (Clark et al., 2012). Second, subjects in false memory studies typically learn their memory is false only days or weeks after coming to believe in it. By contrast, most subjects in these experiments believed in their retracted memories for many years before retracting them. Finally, research shows that people tend to find participating in false memory studies both enjoyable and valuable (Murphy et al., 2020).

Our findings also have implications for our understanding of how common false memories are. The data we report here are at odds with recent claims that false memories are rare and occur only as a consequence of highly suggestive procedures (Brewin et al., 2020). Across four experiments, we found that some 26% to 45% of people have at least one memory they now believe to be false. These proportions are consistent with other studies of retracted memories and provide further support for the idea that misinformation can be readily incorporated into people’s memories (Johnson et al., 1988; Mazzoni et al., 2010). It is possible that some subjects had incorrectly retracted memories of events that really did happen (Scoboria et al., 2014). But people are generally unwilling to invest effort in questioning the accuracy of their memories, so it is unlikely they would have done so without compelling reasons (Nash et al., 2017; Wade et al., 2014). Moreover, because it is often difficult to distinguish real from false memories, it is likely that some people have yet to realize, or may never realize, that they harbor false memories that should be retracted (Johnson et al., 1993). Therefore, our data might actually underestimate the frequency of false memories in the general population.

Taken together, our data show that people think their retracted memories serve both helpful and harmful functions. These results highlight the potential for false memories to have lasting effects on thinking and behavior, even after they have been retracted.

## Supplementary Information

ESM 1 (DOCX 827 kb)

## Data Availability

The preregistrations, materials, and data for all four experiments are available on the Open Science Framework (https://osf.io/q3sjm/). Our ethical approval precludes us from making subjects’ written responses publicly available, but these responses are available on request.

## Footnotes

1 MTurk is an online platform through which people can sign-up to complete short tasks or participate in research studies for small rewards.

2 The pattern does not change when these subjects are included.

3 A pilot study established this prompt elicits a wide belief distribution; see the Supplemental Materials.

4 We used this prompt, rather than yoking the believed memories to the retracted memories as we did in Experiment 3, so we could counterbalance the order in which people provided and rated the two memories.

## Code availability

The analysis script for the four experiments (written in R) is available on the Open Science Framework (https://osf.io/q3sjm/).
