# Supplementary material for: Memories people no longer believe in can still affect them in helpful and harmful ways
Source: Mem Cognit. 2022 Jun 14;50(6):1319–35. doi: 10.3758/s13421-022-01328-9 (PMC9365748; doi:10.3758/s13421-022-01328-9)
Supplement: Supplementary file 1 — (DOCX 827 kb) [file 13421_2022_1328_MOESM1_ESM.docx]

Supplemental Materials: Memories people no longer believe in can still affect them in helpful and harmful ways

# Demographics

|  | Experiment 1 | Experiment 2 | Experiment 3 |
| --- | --- | --- | --- |
| **Mean age (SD)** | 37.10 (12.34) | 37.08 (12.20) | 36.49 (11.40) |
| **Gender**  Woman  Man  Gender diverse | 236 (63%)  138 (37%)  3 (1%) | 235 (61%)  147 (38%)  1 (1%) | 201 (63%)  115 (36%)  5 (2%) |
| **Education**  Did not finish high school  Finished high school  Undergraduate degree  Masters/PhD | 4 (1%)  143 (38%)  180 (48%)  50 (13%) | 3 (1%)  158 (41%)  175 (46%)  47 (12%) | 3 (1%)  121 (38%)  148 (46%)  49 (15%) |
| *Note*. Demographic information was not collected for Experiment 4 to minimize the length of the survey | | | |

# Memory age distributions from Experiments 1 and 2

## Experiment 1


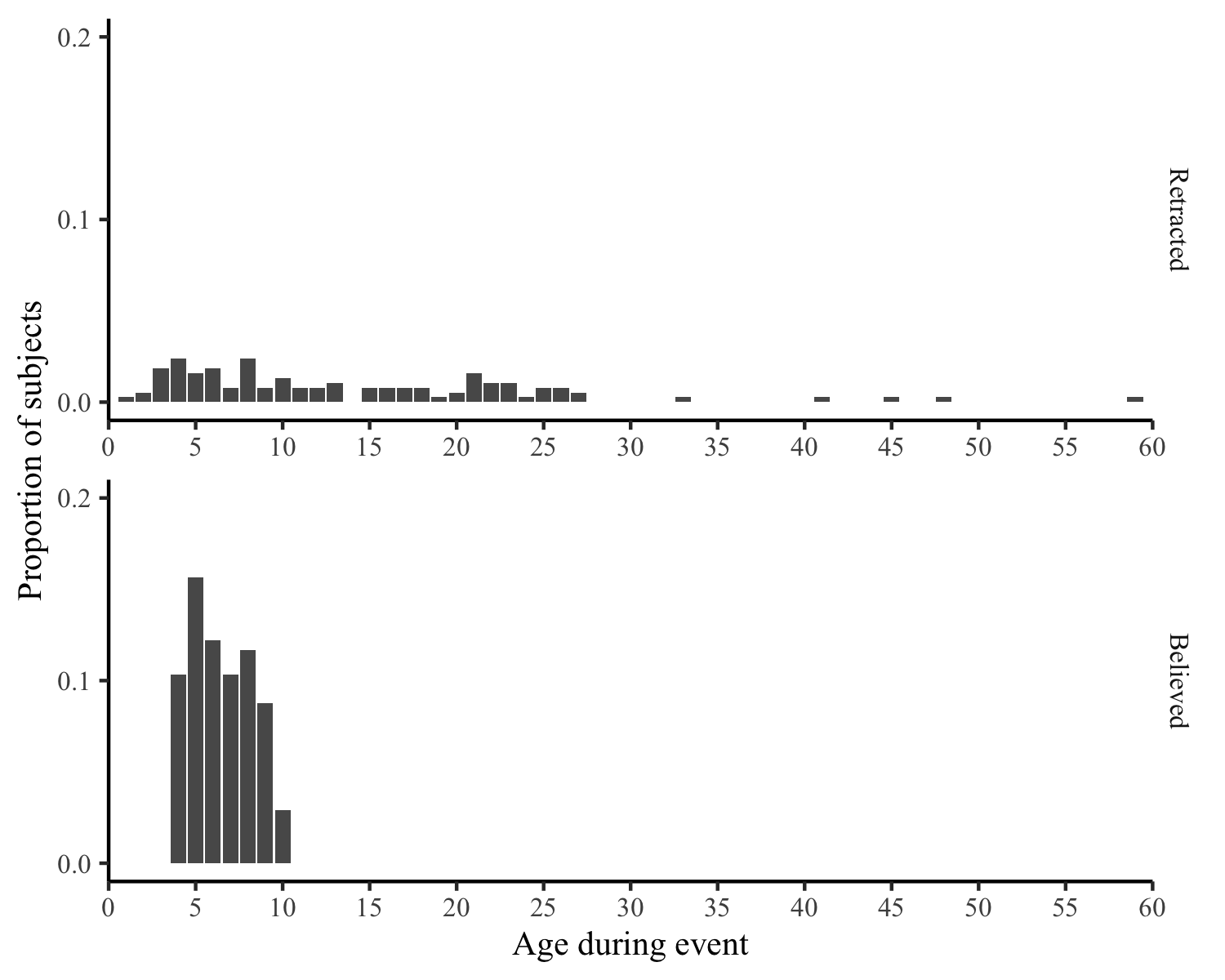


## Experiment 2


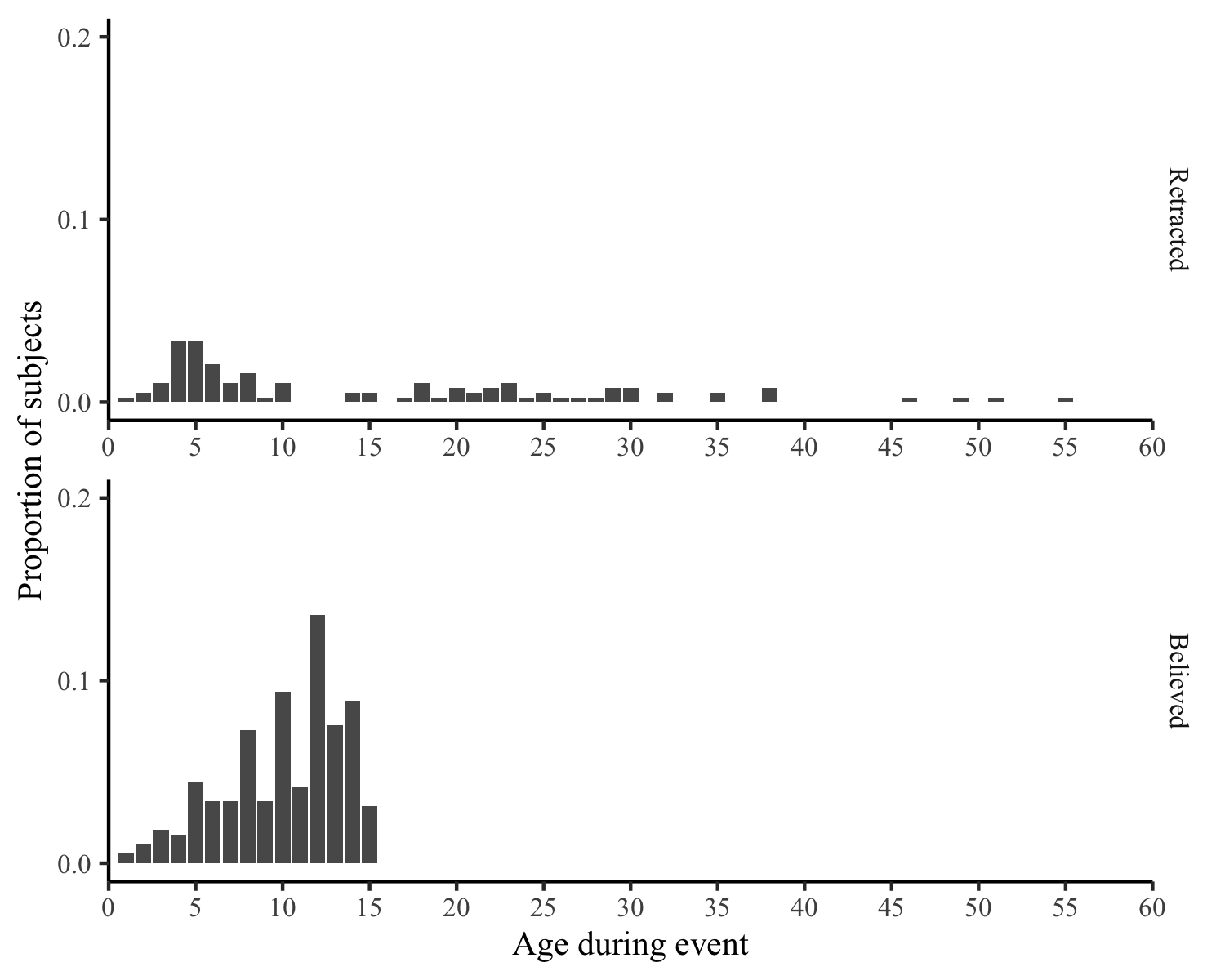


# Phenomenology results from Experiment 3

## Measures

## This memory is significant for my life because it imparts an important message for me or represents a critical juncture or turning point.

## The feelings I experience as I recall the event are positive

## The feelings I experience as I recall the event are negative

## The feelings I experience as I recall the event are intense

## *above items rated from 1 (Not at all) to 7 (To a very high degree)*

## As I remember the event, I feel as though I am reliving it

## As I remember the event, I can see it in my mind

## As I remember the event, I can hear it in my mind

## As I remember the event, I can hear myself or other people talking

## As I remember the event, I know its spatial layout

## As I remember the event, I can recall the setting where it occurred

## *above items rated from 1 (Not at all) to 7 (As if it was happening right now)*

## As I remember the event, I feel that I travel back to the time it happened

## How vivid and clear is your memory for this event?

## If another witness to the event (who you generally trusted) existed and told you a very different account of the event, to what extent could you be persuaded that your memory was wrong?

## *above items rated from 1 (Not at all) to 7 (Completely)*

## Since it happened, I have talked about the event

## Since it happened, I have deliberately thought about the event

## Since it happened, the event has come to me out of the blue, without my trying to think about it

## *above items rated from 1 (Not at all) to 7 (As often as any event in my life)*

## Sometimes people know an event happened to them without being able to actually remember it. As I think about the event, I can actually remember it rather than just knowing that it happened. (*1 = Not at all, 7 = As clearly as if it was happening right now*)

## Results

**
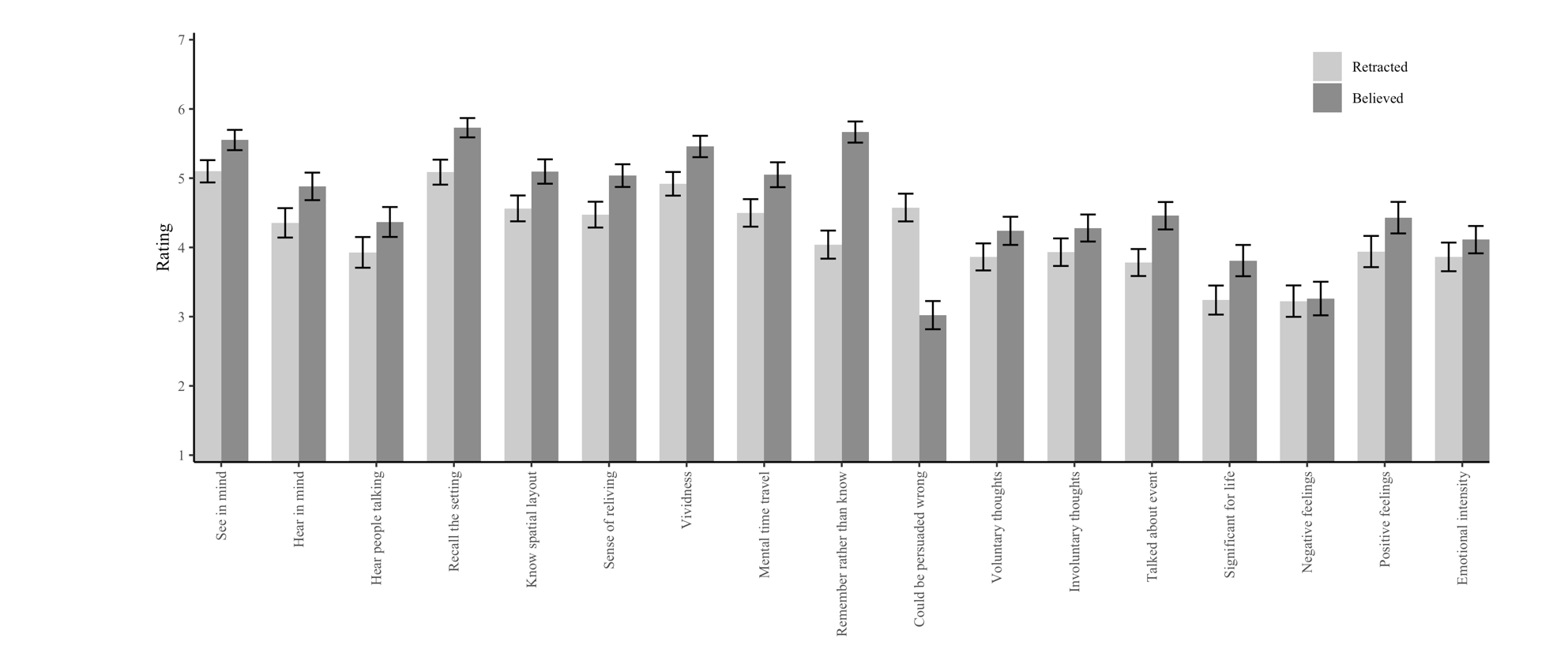
**

# Experiment 3 regression with negative feelings as a predictor

Standardised Beta estimates from the two regressions predicting helpful functions and harmful functions respectively, with reliving, vividness, belief, and negative feelings as predictors.

|  | Helpful function | | Harmful function | |
| --- | --- | --- | --- | --- |
| Predictor | *β* [95% CI] | *p* | *β* [95% CI] | *p* |
| Reliving | -0.21 [0.10, 0.33] | <.001* | -0.03 [-.13, 0.08] | .668 |
| Vividness | 0.09 [-0.04, 0.21] | .154 | 0.01 [-0.10, 0.12] | .901 |
| Belief | 0.27 [0.19, 0.36] | <.001* | 0.12 [0.04, 0.20] | .029* |
| Negative feelings | -0.03 [-0.11, 0.51 | .587 | 0.42[0.35, 0.49] | <.001* |

# Distributions of belief ratings

## Experiment 1

##

*Retracted memories*

*Believed memories*


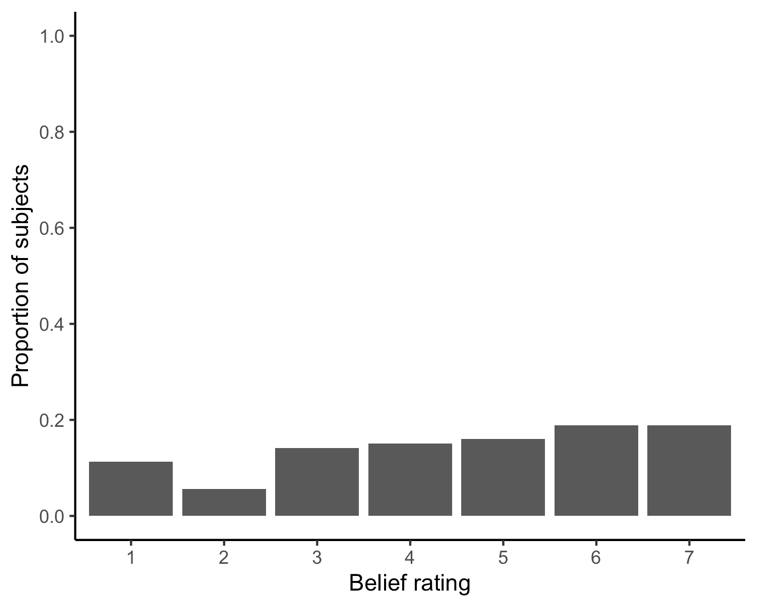

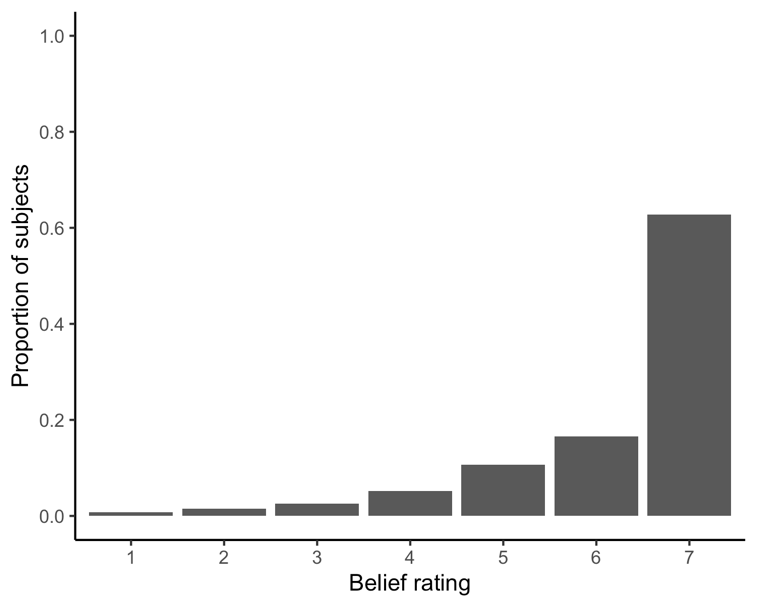


## Experiment 2

*Retracted memories*

*Believed memories*


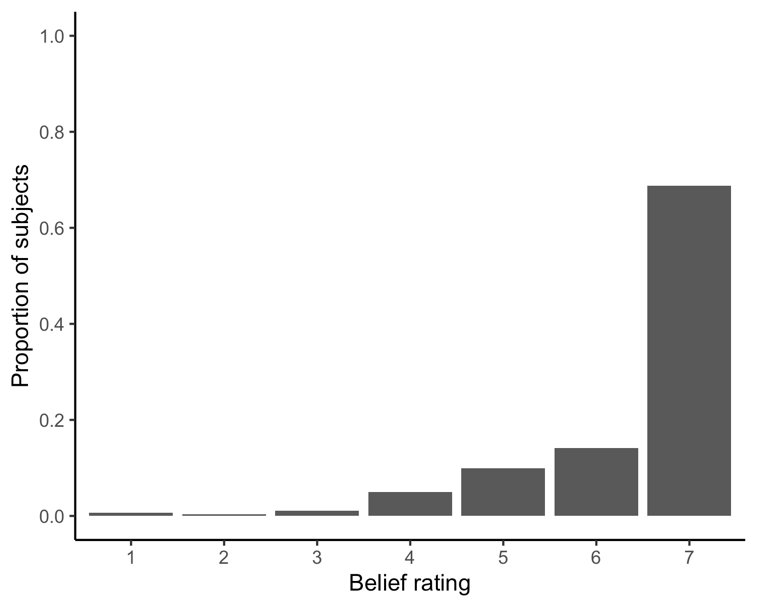

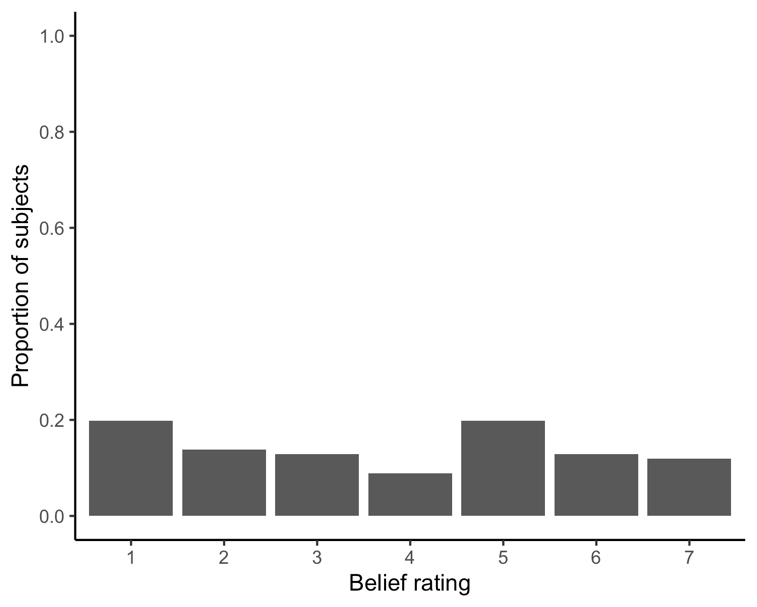


##
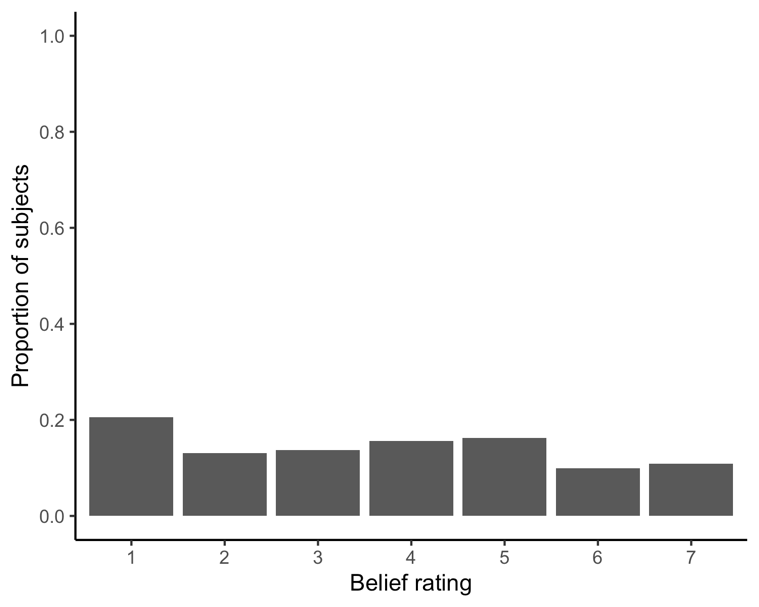

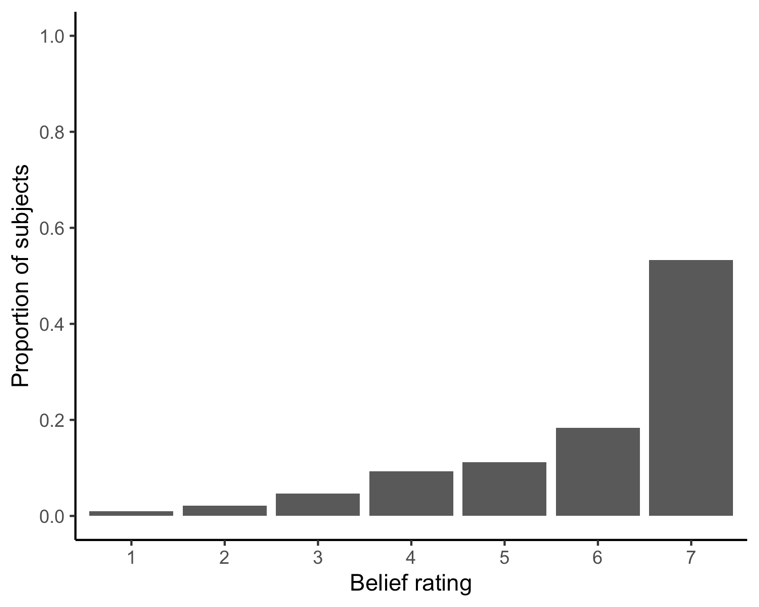
Experiment 3

*Retracted memories*

*Believed memories*

##
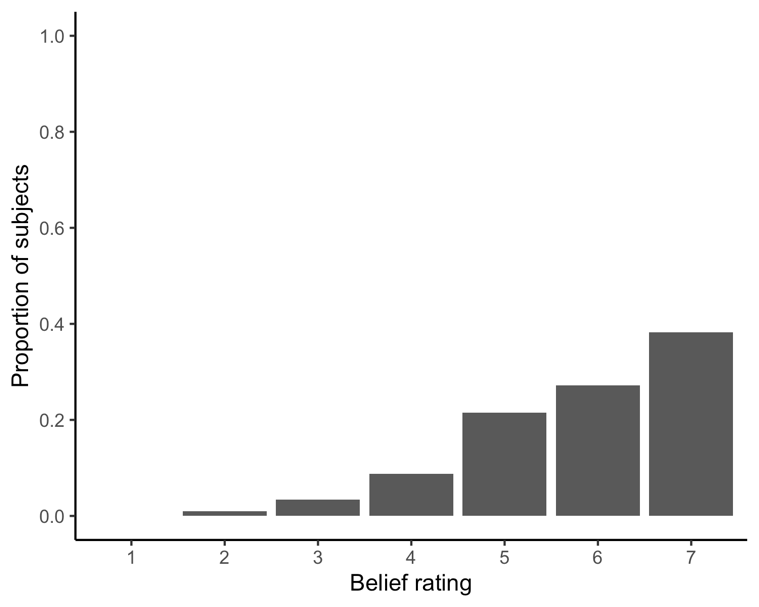

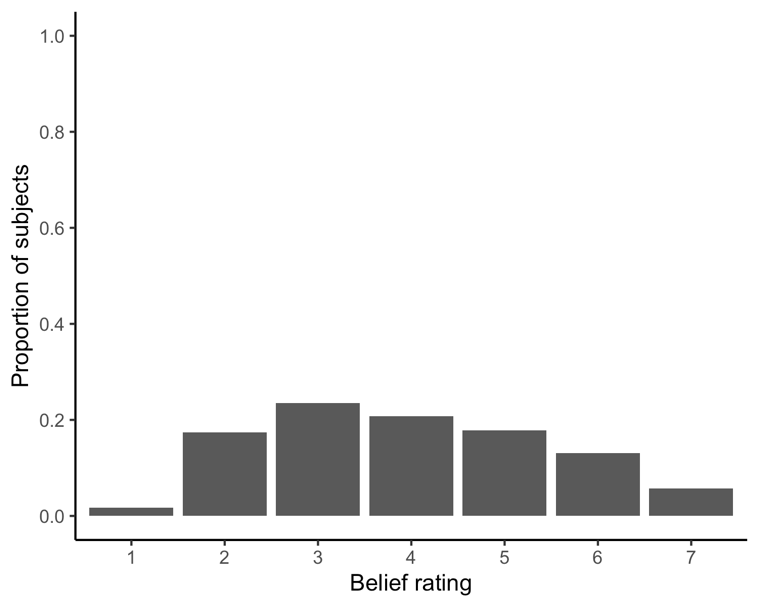
Experiment 4

*Doubted memories*

*Believed memories*

# Pilot study with Doubted Memories Prompt

## Method

### Subjects

We recruited workers from the United States and Canada on Amazon’s Mechanical Turk platform through TurkPrime (Litman et al., 2017). Subjects participated in exchange for Amazon credit. We aimed to collect data until 50 subjects had completed the survey. Because of the way Mechanical Turk interacts with Qualtrics, 52 subjects completed the survey.

### Procedure

First, we provided subjects with a description of a doubted memory: “Sometimes people have doubts about particular memories of their past experiences—that is, they doubt whether the events they remember really happened at all.” Then, we asked subjects to describe the memory they have the most doubts about. Next, subjects rated their belief in the memory on the three items from Rubin et al. (2019). The first of these items was the belief item from Experiments 1-3. The second was “My memory of the event is an accurate reflection of the event as a neutral observer would report it and is not distorted by my beliefs, motives, and expectations” (1 = 100% distorted, 7 = 100% accurate). The third was “Would you be confident enough in your memory of the event to testify in a court of law?” (1 = Not at all, 7 = As much as any memory).

## Results

As the figure below shows, our instructions elicited a wide range of memories that are believed to various degrees (*M* = 4.64, *SD* = 1.62).


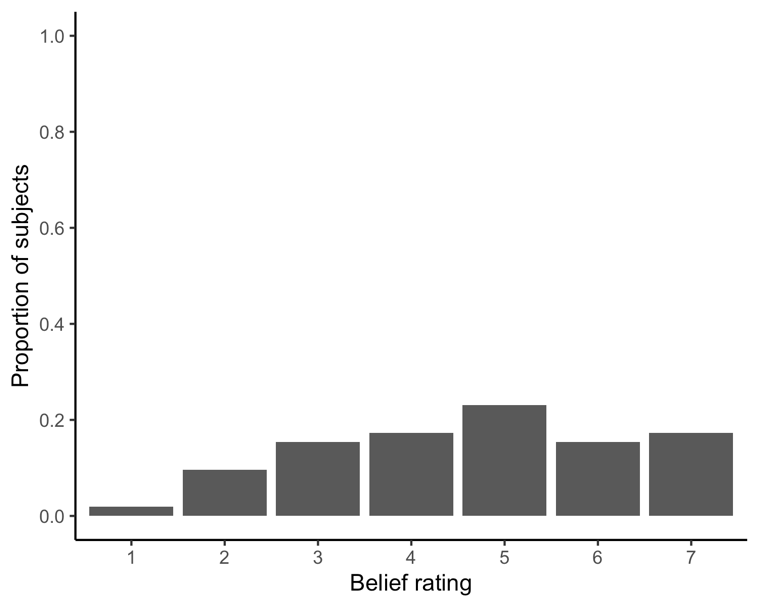


# TALE adapted version

[memory description fed back to subjects]

Please keep the above memory in mind while answering the questions below. We are interested in the extent to which you think back over or talk about the above event in different situations.

For each situation below, your task is to indicate how often, when you think or talk about this event, you do so for the reasons given. Do not hesitate to use any of the points on the scale. If you never think or talk about the event for this reason, circle “Almost never.” Please answer every question. I think back over or talk about this event…

1. when I want to feel that I am the same person that I was before.
2. when I want to remember something that someone else said or did that might help me now.
3. when I hope to find out what another person is like.
4. when I am concerned about whether I am still the same type of person that I was earlier.
5. when I believe that thinking about the past can help guide my future.
6. when I am concerned about whether my values have changed over time.
7. when I want to try to learn from my past mistakes.
8. when I want to develop more intimacy in a relationship.
9. when I need to make a life choice and I am uncertain which path to take.
10. when I want to remember a lesson I learned in the past.
11. when I want to develop a closer relationship with someone.
12. when I want to maintain a friendship by sharing memories with friends.
13. when I am concerned about whether my beliefs have changed over time.
14. when I hope to learn more about another person’s life.
15. when I want to understand how I have changed from who I was before.

*All items rated on the following scale: Almost never, Seldom, Occasionally, Often, Very frequently*

*Self items: 1,4,6,13,15* *Social items: 3,8,11,12,14* *Directive items: 2,5,7,9,10*

# Correlations between age during event, length believed, and functions

## Experiment 1

We found no significant relationship between how long people believed a retracted memory and how helpful that memory was, *r*(104) = -0.18, 95% CI [-0.36, 0.01]. Likewise, we found no relationship between how long people believed a retracted memory and how harmful that memory was, *r*(104) = -0.18, 95% CI [-0.36, 0.01].

We also found no significant relationship between long ago a memory was retracted and how helpful that memory was, *r*(104) = -0.14, 95% CI [-0.32, 0.05]. Likewise, we found no relationship between how long people believed a retracted memory and how harmful that memory was, *r*(104) = -0.12, 95% CI [-0.31, 0.07].

We did, however, find that the older people were when the retracted event “occurred,” the more that memory tended to serve helpful and harmful functions, *r*_helpful_(104) = 0.31, 95% CI [0.13, 0.47]; *r*_harmful_(104) = 0.29, 95% CI [0.11, 0.46].

## Experiment 2

We found no significant relationship between how long people believed a retracted memory and how helpful that memory was, *r*(99) = -0.03, 95% CI [-0.22, 0.17]. Likewise, we found no relationship between how long people believed a retracted memory and how harmful that memory was, *r*(99) = 0.01, 95% CI [-0.19, 0.20].

We also found no significant relationship between long ago a memory was retracted and how helpful that memory was, *r*(99) = -0.16, 95% CI [-0.35, 0.03]. Likewise, we found no relationship between how long people believed a retracted memory and how harmful that memory was, *r*(99) = -0.13, 95% CI [-0.32, 0.07].

In contrast to Experiment 1, we found no significant relationship between the age at which a retracted event “occurred” and how helpful or harmful that memory was, *r*_helpful_(99) = 0.06, 95% CI [-0.13, 0.26]; *r*_harmful_(99) = 0.12, 95% CI [-0.08, 0.30].

## Experiment 3

We found that the longer people had believed a retracted memory, the less helpful that memory tended to be, *r*(319) = -0.19, 95% CI [-0.30, -0.09]. ]. Likewise, the longer people had believed a retracted memory, the less harmful that memory tended to be, *r*(319) = -0.21, 95% CI [-0.31, -0.10]. One possible explanation for these findings is that memories that were believed for long periods also tended to be memories that occurred at very young ages—perhaps, then, these memories tend to be less relevant to people’s current thinking and behavior than retracted memories that “occurred” more recently.

We found that the more time had passed since a memory was retracted, the less helpful that memory tended to be, *r*(319) = -0.13, 95% CI [-0.24, -0.02]. Likewise, the more time had passed since someone retracted a memory, the less harmful that memory tended to be, *r*(319) = -0.14, 95% CI [-0.25, -0.03].

Consistent with Experiment 1, we found that the older people were when the retracted event “occurred,” the more that memory tended to serve helpful and harmful functions, *r*_helpful_(319) = -0.19, 95% CI [0.09, 0.30]; *r*_harmful_(319) = 0.11, 95% CI [0.01, 0.22].
